# Protocol for estimating the contrast transfer function and absolute tilt angle offset for cryo-electron tomography using CTFMeasure

**DOI:** 10.1016/j.xpro.2024.103550

**Published:** 2025-01-22

**Authors:** Ranhao Zhang, Yuan Shen, Xueming Li

**Affiliations:** 1 Key Laboratory for Protein Sciences of Ministry of Education, School of Life Sciences, Tsinghua University, Beijing 100084, China; 2 State Key Laboratory of Membrane Biology, School of Life Sciences, Tsinghua University, Beijing 100084, China; 3 Tsinghua-Peking Joint Center for Life Sciences, Beijing 100084, China; 4 Beijing Frontier Research Center for Biological Structure, Beijing 100084, China; 5 School of Life Sciences, Tsinghua University, Beijing 100084, China; 6 Department of Electronic Engineering, Tsinghua University, Beijing 100084, China; 7 Beijing National Research Center for Information Science and Technology, Tsinghua University, Beijing 100084, China

**Keywords:** Cell Biology, Microscopy, Cryo-EM

## Abstract

Contrast transfer function (CTF) estimation is essential to the data processing workflow of cryo-electron tomography (cryoET). Here, we present a protocol for CTF estimation of the cryoET tilt series with CTFMeasure. CTFMeasure can estimate the CTF parameters together with the absolute tilt angle offset of the sample. We describe steps for configuring the input files and estimating the CTF parameters and the absolute tilt angle offset of the input tilt series. We then detail procedures for visualizing the power spectra and analyzing the output files.

For complete details on the use and execution of this protocol, please refer to Zhang et al.^1^

## Highlights

• Instructions for CTF and absolute tilt angle offset estimation with CTFMeasure
• Visualizing the output power spectra of the tilt series for validation
• Generating the output files with the estimation outcomes for further processing

## Abstract (editor highlights)

Publisher’s note: Undertaking any experimental protocol requires adherence to local institutional guidelines for laboratory safety and ethics.

## Graphical abstract

**Figure:**
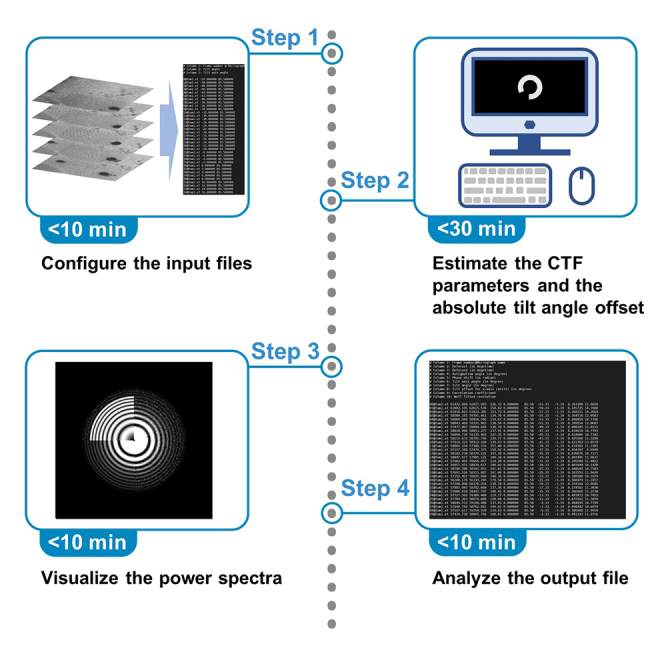

## Before you begin

The protocol below describes the estimation of CTF parameters and the absolute tilt angle offset. A dataset “tom1.st” in EMPIAR-10700^2^^,^^3^ is used in this protocol and can be downloaded from https://www.ebi.ac.uk/empiar/EMPIAR-10700/. The “.st” file is an MRC file containing all the micrographs of a tilt series, which are arranged in ascending order according to their tilt angles. The “.st” file is usually required by IMOD,^4^ and can be generated by the IMOD’s subroutine “newstack” that can combine multiple micrographs into a single MRC stack file. Nevertheless, CTFMeasure does not require such a stack file, and, alternatively, uses a micrograph list to describe the input micrographs of a tilt series. That is, both a combined “.st” file and a series of individual micrograph files are accepted for input. The details of creating the micrograph list file for input will be shown in the section “configure the input files” below.

### Hardware preparation

A computer with at least 32 GB of RAM is required. A CPU with at least 10 cores is suggested. GPUs are not required.

***Note:*** The protocol below is carried out on a Linux operation system.

### Software preparation

**Timing: <0.5 h**

1. Option 1: Compile CTFMeasure from source code.
  a. Download the installation package of CTFMeasure from https://thuem.net/software/ctfmeasure/download.html. The current version of CTFMeasure is v1.4.0. The following example is tested based on this latest version, which corresponds to the package “CTFMeasure_v1.4.0.tar.gz”.
    ***Note:*** If the clickable link on the webpage fails to initiate the download, you can try right-clicking on the link and selecting “Save link as…”.
  b. Decompress the installation package.
    >tar -zxvf CTFMeasure_v1.4.0.tar.gz
    >cd ctfmeasure-master
  c. Install the dependencies. The dependencies include FFTW3,^5^ NLopt,^6^ and Eigen.^7^
    >cd external
    >./build-fftw.sh
    >./build-nlopt.sh
    >cd ..
    ***Note:*** CMake is required to compile NLopt.
  d. Install CTFMeasure.
    >make clean
    >make
    **CRITICAL:** It is important to ensure that the installation of CTFMeasure and its dependencies is successful. Please run the following command. The output will show the help information on the screen (Figure 1), which indicates that CTFMeasure has been installed correctly.
    .
    >./bin/CTFMeasure

**Figure 1:**
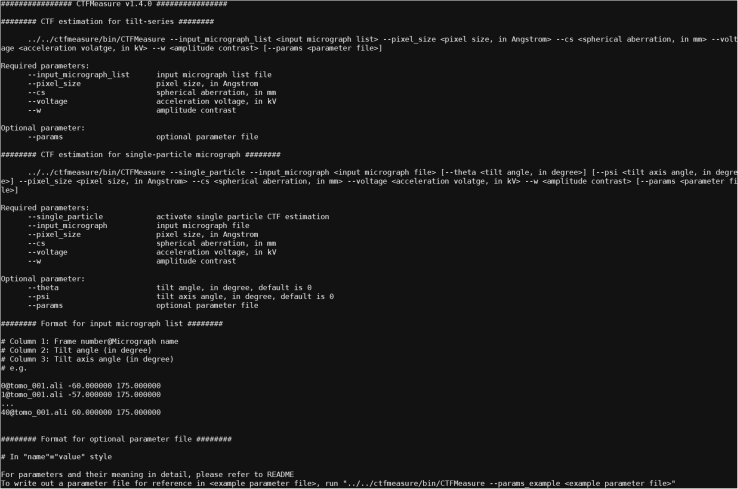
Help information from CTFMeasure When running CTFMeasure without specifying arguments, or missing essential arguments for running, CTFMeasure will display the help information on the screen.
2. Option 2: Use pre-compiled binaries.
  a. Download the pre-compiled binaries of CTFMeasure named “CTFMeasure_v1.4.0_bin.tar.gz” from https://thuem.net/software/ctfmeasure/download.html.
  b. Decompress the package.

>tar -zxvf CTFMeasure_v1.4.0_bin.tar.gz

>cd ctfmeasure-master

***Note:*** The binaries have been tested on Centos and RedHat Linux systems.

3. Setup the environment variables in bash shell.

>cd bin

>export PATH=$PATH:`pwd`

>cd ..

***Note:*** If you are using different shell environment, please change “export” to the corresponding command for that environment. If this step is skipped, CTFMeasure can still be run with its absolute path, namely, “$ROOTPATH/CTFMeasure”. You can also add the program binary folder into your environment configuration script (e.g., ∼/.bashrc) to automatically load the setting.

### Data preparation

**Timing: <0.5 h**

4. Download the parameter file from https://thuem.net/website/CTFMeasure/Examples/para_10700.zip and decompress the zip file.
5. Place the tilt series file “tom1.st” and the parameter file “para_10700.conf” in the same directory.

## Key resources table

**Table:** 

REAGENT or RESOURCE	SOURCE	IDENTIFIER
**Deposited data**		

Dataset of human DLD-1 cells	Schuller et al.^2^	EMPIAR: 10700

**Software and algorithms**		

CTFMeasure	Zhang et al.^1^	https://thuem.net/software/ctfmeasure/overview.html
IMOD	Kremer et al.^4^	https://bio3d.colorado.edu/imod/

## Step-by-step method details

In this section, we describe the steps to estimate the CTF parameters and the absolute tilt angle offset of “tom1.st” in EMPIAR-10700.

### Configure the input files

**Timing: <10 min**

**Figure 2:**
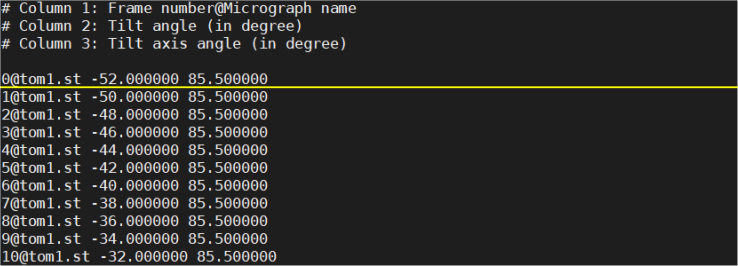
Demonstration of the input micrograph list file Each line represents the information of one micrograph in the tilt series for estimation. The first column is in a “Frame number@Micrograph name” format. The frame number starts from 0. The micrograph name is the name of the MRC file which contains the micrograph. The second column is the tilt angle of the micrograph in degree. The third column is the direction angle of the stage tilt axis in degree.

In this section, we describe the steps to configure the input data files for subsequent processing. CTFMeasure uses a micrograph list file that combines all the micrographs of a tilt series to manage the input data. The micrograph list file is a plain text file of a list specifying the micrographs for estimation (Figure 2). Each line represents the information of one micrograph in the tilt series, and contains no less than three columns. The first three columns are required in the current version (v1.4.0). The columns beyond the third will be ignored by the program currently and may be added in future functional extensions. The first column has a “Frame number@Micrograph name” format. The frame number starts from 0, which indicates the frame number in an MRC file. The micrograph name is the micrograph filename with the absolute path or the path relative to the input micrograph list file. The second column is the stage tilt angle of the micrograph in degree, and the third column is the direction angle of the stage tilt axis in degree.

For the tilt series file “tom1.st” used in this example, all the micrographs are stored in this MRC stack file. Users can either manually generate a micrograph list file or use the following method.

1. Go to the work path that contains the tilt series file “tom1.st” and the parameter file “para_10700.conf”.
2. Generate the text file containing the raw tilt angles.

>seq -52 2 68 >> tom1.rawtlt

***Note:*** The test tilt series “tom1.st” was collected with sample tilt angles from −52° to 68° using a step size of 2°. The generated plain text file “tom1.rawtlt” contains many lines. Each line contains one number, representing the corresponding tilt angle of each micrograph in the tilt series. The order of the angle value in “tom1.rawtlt” must be the same as the order of the images in “tom1.st”.

***Note:*** “seq” is a Linux system command.

***Note:*** You can also generate the “rawtlt” file by typing in the tilt angles manually through a text editor. Please make sure that each line in the “rawtlt” file contains one angle value. Please also make sure that the file “tom1.rawtlt” and the tilt series file “tom1.st” are in the same directory.

3. Generate the input micrograph list file.

**Figure 3:**
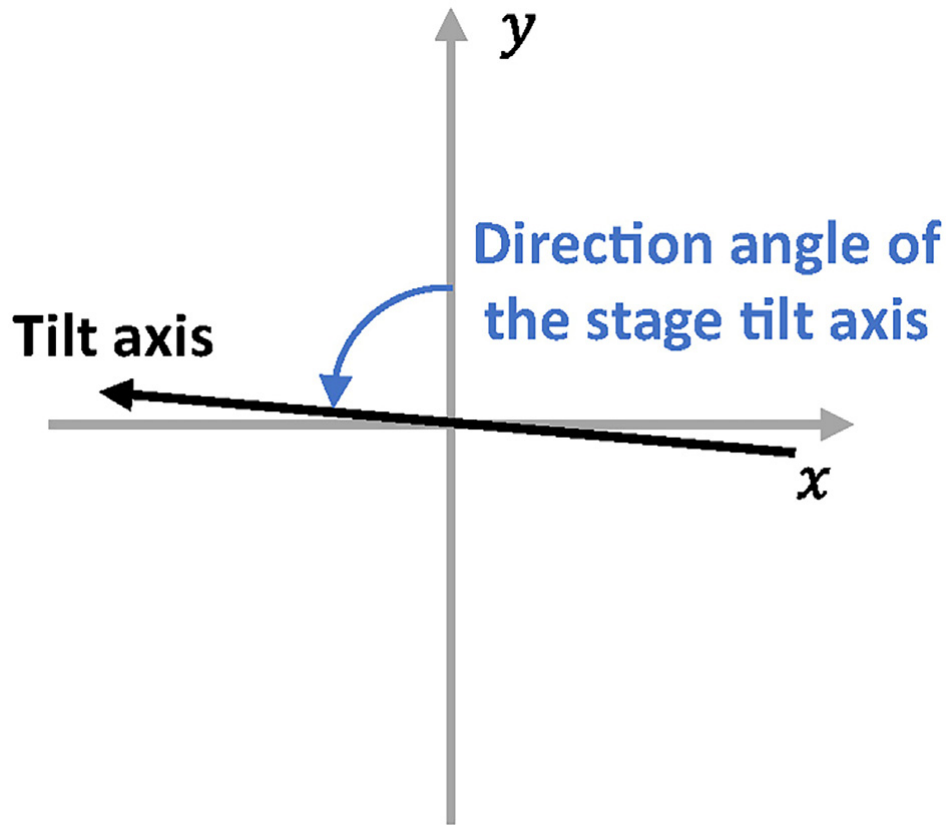
Graphical illustration of the direction angle of the stage tilt axis ψ The black line with an arrow represents the tilt axis. The arrow points the positive direction. The angle between the positive y-axis and the positive tilt-axis is the direction angle of the stage tilt axis ψ. A positive value means rotating the micrograph counter-clockwise to bring the y-axis to the tilt-axis.

>stack_to_list tom1.st tom1.rawtlt 85.5 tom1.list

***Note:*** The program “stack_to_list” is in the package and compiled together with CTFMeasure. It generates an input micrograph list file “tom1.list” based on files “tom1.st”, “tom1.rawtlt”, and the direction angle of the stage tilt axis. In CTFMeasure, the direction angle of the stage tilt axis is the angle between the positive y-axis of the micrograph and the positive tilt-axis of the sample holder, which takes the same definition as the “Tilt axis angle” specified in the “.mdoc” file when collecting data using SerialEM.^8^ A positive value means rotating the micrograph counter-clockwise to bring the positive y-axis of the micrograph to the positive tilt-axis of the sample holder (Figure 3). The direction angle of the stage tilt axis of the test tilt series “tom1.st” is 85.5°.

***Note:*** If you cannot find the command “stack_to_list”, see troubleshooting 1.

4. Check the existence and the format of the parameter file (Table 1).

**Table 1:** The names, values, and description of the parameters in the parameter file

Category	Name	Value	Description
Basic parameters	j	10	Number of threads for parallelization
Parameters for CTF estimation	N_zeros	8	Number of CTF zeros for background estimation and CTF estimation
	resolution_max	10.0	Maximum resolution for fitting (in Angstrom)
	resolution_min	30.0	Minimum resolution for fitting (in Angstrom)
	resolution_adaptive	1	Use adaptive resolution range for fitting (0-Not use; 1-Use. If set to 1, the maximum and minimum resolution for fitting will be adjusted according to the given “N_zeros” and the estimated CTF parameters in the initial estimation step.)
	it	3	Number of iterations in the iterative per-micrograph estimation step
Advanced parameters	box_conv	25	Convolution box size for background estimation
	N_avg	1	Number of adjacent micrographs averaged during the initial estimation step
	optimize_with_average	0	Fit with the average power spectrum during estimation (0-Use the original power spectrum for fitting; 1-Use the average power spectrum for fitting)
	tight_blocks	1	Ignore the outermost blocks to neglect the boundary of the micrographs (0-Using all blocks; 1- Using tight blocks, ignoring the outermost blocks)
Parameters for angle estimation	pre_offset_estimation	1	Apply absolute tilt angle offset estimation during the initial estimation step (0-Not apply; 1-Apply)
	skip_offset_estimation	0	Skip estimating the absolute tilt angle offset during the iterative per-micrograph estimation step (0-Not skip; 1-Skip)
	skip_offset_refinement	0	Skip refining the absolute tilt angle offset during the iterative per-micrograph estimation step (0-Not skip; 1-Skip)
	skip_offset_estimation_x	0	Skip estimating the off-plane tilt angle of the tilt axis (x-tilt) during the iterative per-micrograph estimation step (0-Not skip; 1-Skip)
	skip_offset_refinement_x	0	Skip refining the off-plane tilt angle of the tilt axis (x-tilt) during the iterative per-micrograph estimation step (0-Not skip; 1-Skip)

>cat para_10700.conf

***Note:*** The parameter file “para_10700.conf” is a plain text file containing the parameters for estimation. In the parameter file, each line must only contain one parameter with the format of “{parameter name}={parameter value}”. There must be no spaces before and after the “=” sign. The parameters in the parameter file “para_10700.conf” are described in Table 1. To generate a template parameter file for reference, please run the following command. This command will generate a parameter file named “para_example.conf” under the current path. For detailed description of a full list of all parameters, please refer to https://thuem.net/software/ctfmeasure/ctfestimation.html#parameter-file-optional.

> CTFMeasure --params_example para_example.conf

***Note:*** “cat” is a Linux system command.

### Estimate the CTF parameters and the absolute tilt angle offset

**Timing: <30 min**

In this section, we estimate the CTF parameters and the absolute tilt angle offset of the input tilt series.

5. Run CTFMeasure.

>CTFMeasure --input_micrograph_list tom1.list --pixel_size 3.418 --cs 2.7 --voltage 300 --w 0.07 --params para_10700.conf

***Note:*** On a workstation with 2 Intel(R) Xeon(R) Gold 6132 CPUs, the above process took ∼20 min.

***Note:*** Upon a successful running, CTFMeasure will output three data files and some running information on the screen.

The three output files include: an MRC file of the average power spectrum of the tilt series (“tom1_avg.mrc”), an MRC file of the power spectrum of each micrograph in the tilt series (“tom1_diag.st”), and a plain text file containing the estimated CTF and angle parameters (“tom1_ctf.txt”). The three distinct output files are identifiable through their specific suffixes: “_avg.mrc”, “_diag.st”, and “_ctf.txt”. These suffixes are automatically appended by CTFMeasure to the primary filename of the input micrograph list file by default.

The screen output shows three major steps.

Before the estimation, the program prints all the parameter values to the screen in the “Read parameters” section.

During the estimation, firstly, the program prints the tilt angles and the average densities of the corresponding micrographs on the screen, and then estimates the absolute tilt angle offset of the input tilt series, termed “Estimated global tilt offset” on the screen. Then, a coarse defocus value without considering astigmatism is estimated by exhaustive search. The defocus values and their corresponding correlation coefficients (termed “cc”) are printed on the screen.

Secondly, the “Tilt-series joint CTF estimation” is applied to estimate the average defocus of the tilt series with astigmatism. The program prints the defocus values and the corresponding correlation coefficients at different astigmatism angles.

Thirdly, the “Per-micrograph CTF refinement” step iteratively optimizes the defocus values for each micrograph and the angle parameters of the tilt series. The estimated defocus values and the corresponding correlation coefficients of each micrograph are printed on the screen. The “tilt axis azimuth” and the “tilt offset for x-axis” in the output both represent the off-plane tilt angle of the tilt axis (x-tilt). The absolute tilt angle offset shown during refinement is the offset from the previous iteration. The final value of the absolute tilt angle offset is calculated as the sum of the opposite number of the offset value from the initial estimation and the offset values from each iteration.

***Note:*** If you cannot find the command “CTFMeasure”, see troubleshooting 1.

***Note:*** If you get errors in running CTFMeasure, see troubleshooting 2.

***Note:*** If you find that the computation time of CTFMeasure is too long, see troubleshooting 3.

***Note:*** If CTFMeasure crashes during execution, see troubleshooting 4.

### Visualize the power spectra

**Timing: <10 min**

In this section, we visualize the output power spectra of the tilt series to check the quality of the data and the correctness of the estimation. 3dmod in IMOD^4^ is used in this protocol to visualize MRC files. You can also use other software for visualization.

**Figure 4:**
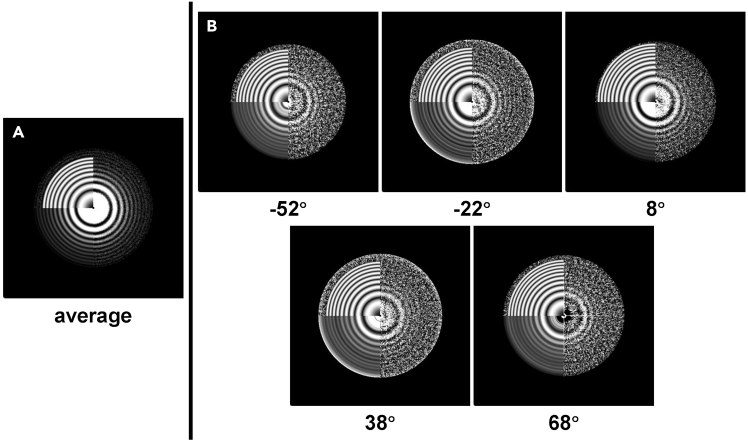
Visualization of the power spectra The right half of each image shows the experimental power spectrum. The bottom left corner shows the rotational average of the power spectrum. The theoretical CTF calculated with the estimated defocus is inset in the top left corner of the power spectrum. (A) The average power spectrum of the tilt series. (B) The power spectra of the selected micrographs. The raw tilt angle of the micrograph is listed below the power spectrum.

6. Visualize the average power spectrum of the tilt series (Figure 4A).

>3dmod tom1_avg.mrc

7. Visualize the power spectrum of each micrograph in the tilt series (Figure 4B).

>3dmod tom1_diag.st

***Note:*** “tom_avg.mrc” and “tom1_diag.st” are both MRC files.

***Note:*** If the experimental Thon rings in the power spectra do not match the theoretical Thon rings computed with the estimated defocus, see troubleshooting 5.

### Analyze the output file

**Timing: <10 min**

In this section, we describe how to understand the estimated results in the output text file.

**Figure 5:**
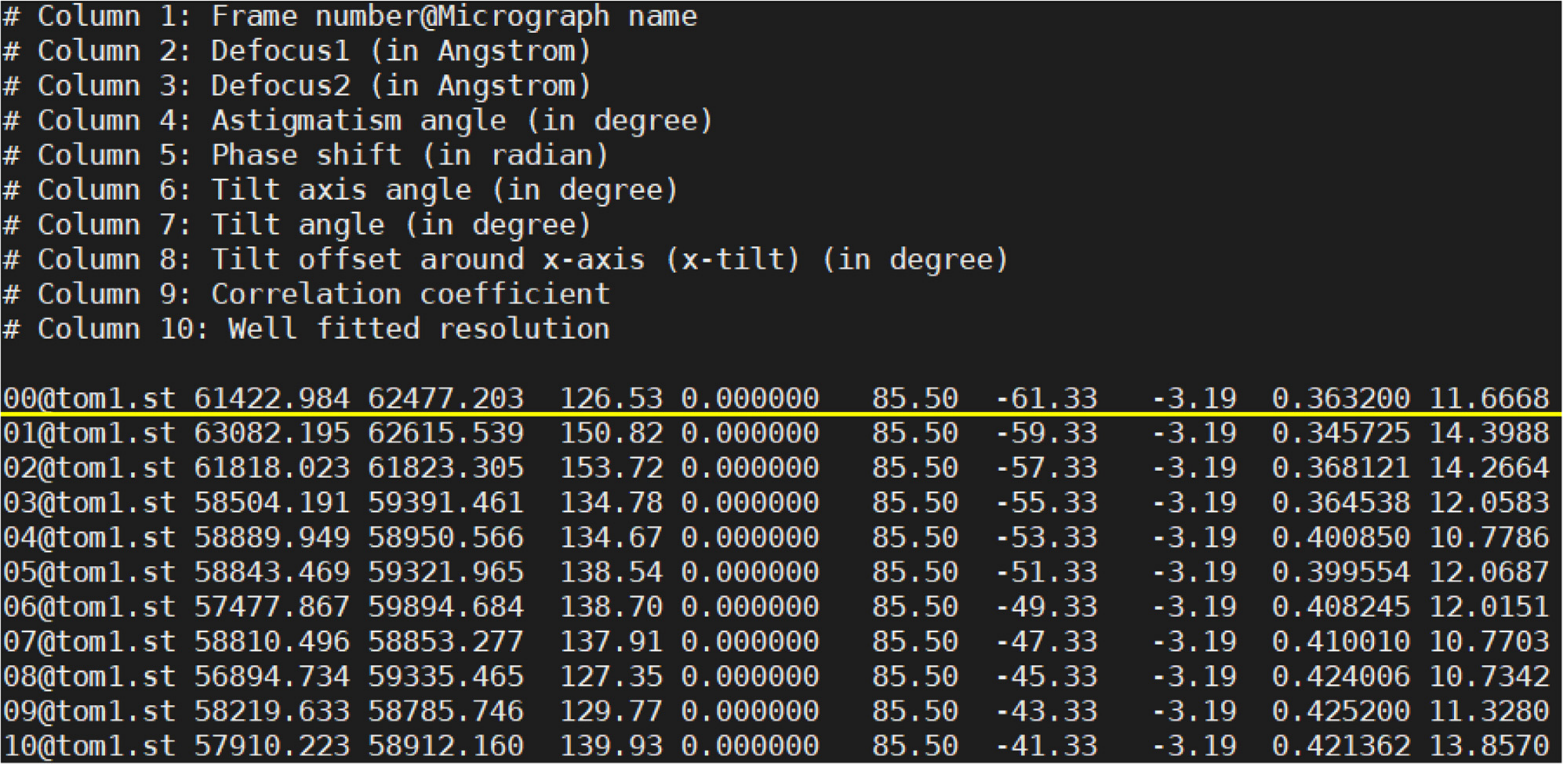
Demonstration of the output file Each line represents the information of one micrograph in the tilt series for estimation. Each line contains ten columns, representing “Frame number@Micrograph name”, the defocus value along the major axis (in Angstrom), the defocus value along the minor axis (in Angstrom), the astigmatism angle (in degree), the phase shift (in radian), the direction angle of the tilt axis (in degree), the tilt angle (in degree), the off-plane tilt angle of the tilt axis (x-tilt, in degree), the correlation coefficient between the experimental power spectrum and the power spectrum calculated with the estimated defocus, and the resolution of the power spectrum fitting (in Angstrom).

8. Check the estimated defocus in the output file (Figure 5).

>cat tom1_ctf.txt

***Note:*** The output file “tom1_ctf.txt” is a plain text file with ten columns. The first column specifies the name of the micrograph. The second to the fourth columns specify the defocus value along the major axis (in Angstrom), the defocus value along the minor axis (in Angstrom), and the astigmatism angle (in degree), respectively. These three columns show the results of CTF estimation. The sixth to the eighth columns specify the direction angle of the tilt axis (in degree), the tilt angle (in degree), and the off-plane tilt angle of the tilt axis (x-tilt, in degree), respectively. These three columns show the results of absolute tilt angle offset estimation. The tenth column specifies the resolution of the power spectrum fitting (in Angstrom), which can be used as a metric for assessing the quality of estimation.

***Note:*** If the resolution of the power spectrum fitting (the tenth column in the output file) is worse than 30 Å, see troubleshooting 6.

***Optional:*** Convert the output file to the CTFFIND4 format for further processing.

>ctfmeasure_to_ctffind4 tom1_ctf.txt defocus_file.txt tom1.tlt

>cat defocus_file.txt

***Note:*** The program “ctfmeasure_to_ctffind4” is compiled together with CTFMeasure. A plain text file “defocus_file.txt” containing the defocus of the micrographs in the CTFFIND4 format will be generated, together with a plain text file “tom1.tlt” containing the tilt angles of the micrographs. The CTFFIND4-format output can be used in further processing, such as tomogram reconstruction with NovaCTF^9^ and subtomogram averaging with RELION.^10^^,^^11^

***Note:*** If you cannot find the command “ctfmeasure_to_ctffind4”, see troubleshooting 1.

## Expected outcomes

After a successful running, three output files (“tom1_avg.mrc”, “tom1_diag.st”, and “tom1_ctf.txt”) should be generated and show reasonable results as discussed in the notes of the sections above. The typical results of the examined dataset “tom1.st” are shown in Figures 4 and 5.

## Limitations

When applying the protocol to other datasets, the parameters need to be tuned to optimize the estimation. Running CTFMeasure with the default parameters usually yields a reasonable result, but it may not be optimal.

## Troubleshooting

### Problem 1

Cannot find the command “stack_to_list” (related to Step 3), “CTFMeasure” (related to Step 5), or “ctfmeasure_to_ctffind4” (related to Step 8).

### Potential solution

Please check that CTFMeasure is installed successfully. “stack_to_list” and “ctfmeasure_to_ctffind4” are two programs compiled together with CTFMeasure. If you did not add the path of CTFMeasure to the environment variables (Step 3 in “before you begin”), please run these programs with the absolute path, namely, “$ROOTPATH/stack_to_list” in Step 3, “$ROOTPATH/CTFMeasure” in Step 5, and “$ROOTPATH/ctfmeasure_to_ctffind4” in Step 8.

### Problem 2

Get errors in running CTFMeasure (related to Step 5).

### Potential solution

Make sure to run CTFMeasure with the arguments “--input_micrograph_list”, “--pixel_size”, “--cs”, “--voltage”, and “--w”. If any of the above arguments are missing, CTFMeasure will fail to run and display the help information on the screen (Figure 1). Besides, “--params” is an optional argument.

### Problem 3

The computing time of CTFMeasure is too long (related to Step 5).

### Potential solution

Setting the parameters “skip_offset_estimation”, “skip_offset_refinement”, “skip_offset_estimation_x”, and “skip_offset_refinement_x” to 0 will enable a more precise estimation of the absolute tilt angle offset and the off-plane tilt angle of the tilt axis (x-tilt), while significantly increasing the computing workload. For a quick estimation, setting any of the four parameters to 1 will reduce the computation and shorten the computing time.

Besides, increasing the parameter “j”, which represents the number of threads for parallelization, will reduce the computation time.

### Problem 4

CTFMeasure crashes during execution (related to Step 5).

### Potential solution

Thera are many possible reasons for crashing. You can check the following.

• The input micrograph list file “tom1.list” exists and follows the format described in Step 3;
• The parameter file “para_10700.conf” exists and follows the format described in Step 4;
• The input tilt series file “tom1.st” exists.

### Problem 5

**Figure 6:**
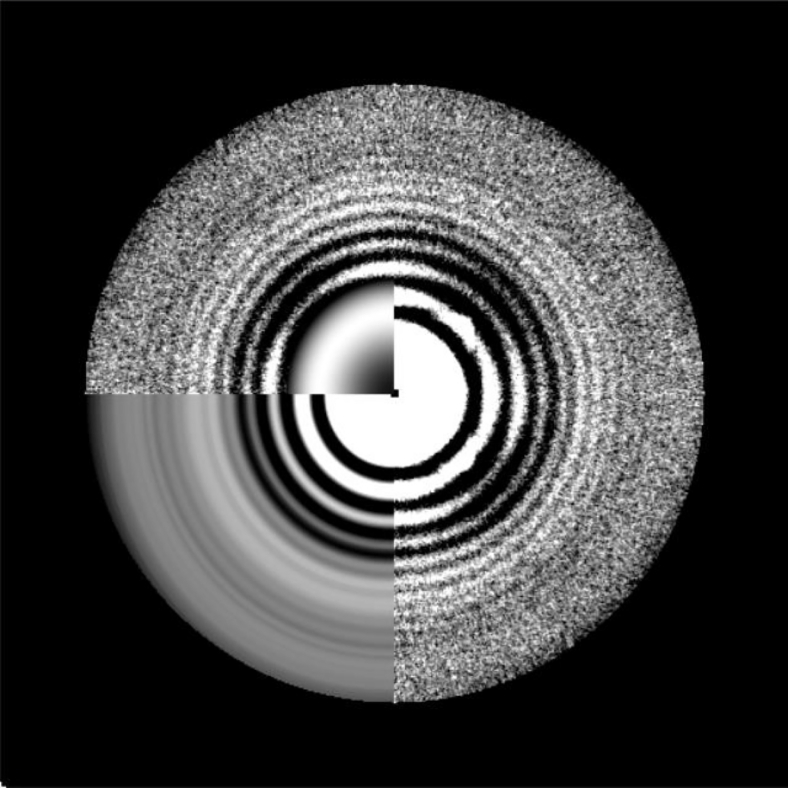
Visualization of the mismatched Thon rings The right half shows the experimental power spectrum and the bottom left corner shows the rotational average of the power spectrum. The theoretical CTF calculated with the estimated defocus, which is inset in the top left corner of the power spectrum, does not match with the experimental Thon rings.

The experimental Thon rings in the power spectra do not match the theoretical Thon rings computed with the estimated defocus (Figure 6, related to Steps 6 and 7).

### Potential solution

This indicates that the CTF estimation may be wrong. We can tune the parameters in the parameter file and rerun CTFMeasure. Sometimes, increasing the parameter “N_zeros” and adjusting the parameters “defocus_min” and “defocus_max” may help. “N_zeros” can be set to the number of visible Thon rings in the average power spectrum of the tilt series. “defocus_max” and “defocus_min” can be set based on the defocus range used during data collection. If there is a large defocus variation among the micrographs in a tilt series, we can set the parameter “per_tilt_estimation” to 1 to estimate the defocus of each micrograph independently.

### Problem 6

The resolution of the power spectrum fitting (the tenth column in the output file) is worse than 30 Å (related to Step 8).

### Potential solution

One possible reason is that the estimation is not accurate. Parameters can be tuned for better estimation accuracy. The commonly used parameters include “defocus_max”, “defocus_min”, “resolution_max”, “resolution_min”, and “N_zeros”. “defocus_max” and “defocus_min” specify the range of defocus values for searching during the initial estimation step. They can be tuned based on the defocus range used during data collection. “resolution_max” and “resolution_min” specify the range of spatial frequencies for fitting during the initial estimation step. “N_zeros” specifies the number of CTF zeros for background estimation and CTF estimation in the coarse overall estimation step and the per-micrograph estimation step. These three parameters can be tuned based on the visible Thon rings in the power spectrum. Specifically, “resolution_min” can be set to the corresponding resolution of the first CTF zero, typically around 30 Å to 50 Å. “resolution_max” is typically set to the highest resolution of the visible Thon rings in the power spectra. “N_zeros” can be set to the number of visible Thon rings in the average power spectrum of the tilt series.

If the merit of the fitting resolution is still worse after tuning the parameters, this may indicate that the data is of low quality.

## Resource availability

### Lead contact

Further information and requests for resources should be directed to and will be fulfilled by the lead contact, Xueming Li (lixueming@tsinghua.edu.cn).

### Technical contact

Technical questions on executing this protocol should be directed to and will be answered by the technical contact, Ranhao Zhang (zrh20@mails.tsinghua.edu.cn).

### Materials availability

This study did not generate new unique reagents.

### Data and code availability

• The dataset used and analyzed in the current study includes EMPIAR-10700.
• The program code and the detailed information about the software usage are available at https://thuem.net.

## Acknowledgments

This work was supported by funds from the National Natural Science Foundation of China (32241023 and 92254306 to X.L.), Tsinghua-Peking Joint Center for Life Sciences, and Beijing Frontier Research Center for Biological Structure. We thank all the users for testing the software. We acknowledge the Tsinghua University Branch of China National Center for Protein Sciences Beijing for providing facility support in computing.

## Author contributions

X.L. and Y.S. initialized the project. R.Z. designed the protocol and performed the experiment. X.L. and R.Z. wrote the manuscript. All the authors revised the manuscript.

## Declaration of interests

The authors declare no competing interests.

## References

[1] Zhang R., Shen Y., Li X. (2024). Tilt-series-based joint CTF estimation for cryo-electron tomography. Structure.

[2] Schuller A.P., Wojtynek M., Mankus D., Tatli M., Kronenberg-Tenga R., Regmi S.G., Dip P.V., Lytton-Jean A.K.R., Brignole E.J., Dasso M. (2021). The cellular environment shapes the nuclear pore complex architecture. Nature.

[3] Iudin A., Korir P.K., Somasundharam S., Weyand S., Cattavitello C., Fonseca N., Salih O., Kleywegt G.J., Patwardhan A. (2023). EMPIAR: the Electron Microscopy Public Image Archive. Nucleic Acids Res..

[4] Kremer J.R., Mastronarde D.N., McIntosh J.R. (1996). Computer Visualization of Three-Dimensional Image Data Using IMOD. J. Struct. Biol..

[5] Frigo M., Johnson S.G. (2005). The Design and Implementation of FFTW3. Proc. IEEE.

[6] Johnson, S.G. (2007). The NLopt nonlinear-optimization package. https://github.com/stevengj/nlopt.

[7] Guennebaud, G., Jacob, B., and others (2010). Eigen v3. http://eigen.tuxfamily.org.

[8] Mastronarde D.N. (2005). Automated electron microscope tomography using robust prediction of specimen movements. J. Struct. Biol..

[9] Turoňová B., Schur F.K.M., Wan W., Briggs J.A.G. (2017). Efficient 3D-CTF correction for cryo-electron tomography using NovaCTF improves subtomogram averaging resolution to 3.4Å. J. Struct. Biol..

[10] Bharat T.A.M., Russo C.J., Löwe J., Passmore L.A., Scheres S.H.W. (2015). Advances in Single-Particle Electron Cryomicroscopy Structure Determination applied to Sub-tomogram Averaging. Structure.

[11] Zivanov J., Otón J., Ke Z., von Kügelgen A., Pyle E., Qu K., Morado D., Castaño-Díez D., Zanetti G., Bharat T.A.M. (2022). A Bayesian approach to single-particle electron cryo-tomography in RELION-4.0. Elife.

